# Stress levels predict substantial improvement in pain intensity after 10 to 12 years in women with fibromyalgia and chronic widespread pain: a cohort study

**DOI:** 10.1186/s41927-019-0072-9

**Published:** 2019-06-24

**Authors:** Anna Bergenheim, Sofia Juhlin, Lena Nordeman, Monica Joelsson, Kaisa Mannerkorpi

**Affiliations:** 1 Institute of Neuroscience and Physiology, Department of Health and Rehabilitation, Physiotherapy Unit, Sahlgrenska Academy, University of Gothenburg, Gothenburg, Sweden; 2 Research and Development Primary Health Care, Region Västra Götaland, Sweden; 3 Närhälsan Rehabilitation Centres, Region Västra Götaland, Sweden

**Keywords:** Fibromyalgia, Chronic pain, Follow-up, Longitudinal

## Abstract

**Background:**

Previous studies of prognosis for women with Fibromyalgia (FM) or chronic widespread pain (CWP) show contradictory results. However, some women appear to improve in pain and other core symptoms over time. There is limited knowledge about predictors of substantial improvement in pain intensity over a longer period of time. The primary objective of this study was to investigate the natural course of pain intensity and distribution of pain over 10 to 12 years in a cohort of 166 women with FM or CWP. Secondarily we wanted to investigate predictors of substantial improvement (*≥*50%) in pain intensity after 10 to 12 years.

**Methods:**

The study is a longitudinal prospective cohort study. 166 women with FM or CWP were followed up after 10 to 12 years. 126 women (76%) participated in the follow-up and completed a battery of questionnaires concerning pain intensity, pain distribution and other physical and mental aspects of health. Differences in symptoms within the cohort over 10 to 12 years and predictors of substantial improvement (*≥*50%) in pain intensity were calculated.

**Results:**

Pain had improved at the 10 to 12 year follow-up (*p* < 0.001) with a mean change of − 9.2 mm (SD 23.3, 95% CI: − 13.3; − 5.0) for pain intensity and − 2.0 sites (SD 4.2, 95% CI: − 2.7; − 1.2) for pain distribution. Nine percent of the 126 women showed an individual moderate improvement in pain intensity while 16% showed a substantial improvement at the follow-up as compared to baseline. Lower symptoms of stress and higher pain intensity at baseline predicted higher probability of reporting at least 50% less pain intensity after 10 to 12 years as compared to baseline.

**Conclusions:**

A majority of women with FM and CWP appear to have a positive course of pain over time, which should be communicated to these patients in health care. Reducing stress levels might contribute to better chances of improvement over time.

**Trial registration:**

Clinicaltrials.gov NCT02872129, registered 06/30/2016.

## Background

The prevalence of chronic widespread pain (CWP) is about 7 to 13% of the population in the western world [1, 2]. CWP has been defined as pain in both body sides, above and below the waist plus axial pain, present for at least 3 months [3]. 1 to 3% of the general population constitute a subgroup of CWP with more severe symptoms [2, 4], fulfilling the tender point criteria for Fibromyalgia (FM) [3]. Besides pain and tenderness, FM and CWP is associated with stiffness, severe fatigue, psychological distress and cognitive difficulties [4–6]. Persons with FM or CWP have also been found to be less physically active and to have lower physical capacity than healthy individuals [7], which increases the risk to develop concomitant disorders.

The results of previous longitudinal studies are contradictory. In a 3 year follow-up [8] of the course of symptoms in 59 women with FM, significant improvements were found for pain intensity and number of tender points and 36% of the women achieved at least 30% improvement in pain intensity. Also, the percentage of patients experiencing widespread pain was reduced from 100 to 63% [8]. Similarly, in a comprehensive follow-up in a general population in Norway, 47% of the persons who reported CWP at baseline had recovered from CWP after 11 years [9]. On the contrary, a longitudinal study including over 3000 participants with FM found no clinically relevant improvements in pain, fatigue or self-rated functional status over 10 years [10]. Other longitudinal studies in patients with FM have not found improvement in pain intensity over 5 to 6 years, but in overall health [11, 12].

To the best of our knowledge, there are no accepted limits representing moderate and substantial improvement in pain intensity in cohort studies of chronic pain. A 15% reduction in pain intensity on a numeric rating scale has been suggested to reflect minimal clinical important difference while a 33% reduction in pain intensity has been suggested to represent a “much better” improvement in cohorts of chronic musculoskeletal pain [13]. Improvements in pain intensity in clinical trials are recommended to be presented as change both on group-level and on individual level as number of persons improving at least 30% (moderate improvement) and 50% (substantial improvement) from baseline for numeric rating scales or Visual Analogue Scales (VAS) [14]. The same limits for moderate and substantial improvement have been used in a large cohort study in persons with FM [15], and were also applied in the present cohort study.

There is limited knowledge of why some patients achieve substantial improvement in pain over time while others do not. A Swedish postal survey showed that higher health related quality of life at baseline in persons with chronic pain increased the chances of no longer having chronic pain after 3 years [16]. On the other hand, factors such as being a woman, having lower education, high Body Mass Index (BMI), higher levels of depression and anxiety, worse sleep, being former smoker or having other concomitant disorders have been found to predict an increased risk of still having CWP after 11 years [9].

Most follow-up studies have been conducted after three to 6 years, and the knowledge of the course of symptoms in FM and CWP over a longer period appear to be scarce.

In the present study, we conducted a follow-up of 166 women with FM or CWP, in order to examine how pain and other health related aspects change over 10 to 12 years, and also if psychological and physical aspects of health could predict the long-term change in pain intensity.

### Objectives

The primary objective of this study was to investigate the natural course of pain intensity and distribution of pain over 10 to 12 years in a cohort of 166 women with FM or CWP. Secondarily, we wanted to investigate predictors of substantial improvement in pain intensity after 10 to 12 years.

Also, we were interested in investigating the natural course over 10 to 12 years in overall health, global fatigue, depression and anxiety, symptoms of stress and health related quality of life.

## Methods

### Study design

A longitudinal cohort study.

### Patients and methods

#### Participants

In the year of 2004–2005, 166 women with FM or CWP recruited in primary care in western Sweden participated in a randomized controlled trial (RCT) called the GAU-study, comparing a patient education program in combination with 20 weeks of pool exercise with a control group participating in a patient education program as only treatment [17]. All participants completed a battery of questionnaires regarding symptoms and health and performed tests of physical function at baseline. The impact of a 20 week intervention was considered to be negligible after 10 to 12 years, thus the whole cohort of 166 women were eligible for recruitment in the present follow-up of natural course of symptoms over time.

##### Inclusion criteria

The 166 women with FM or CWP who participated in the baseline examination of the GAU study 2004–2005 [17] were invited to participate in the present study. The inclusion criteria of the GAU study [3] were women with FM or CWP, in the age range 18–60 years. CWP was classified as pain above and below the waist, at the right and left side of the body and axial pain for at least 3 months. FM was classified as having CWP in combination with pain at manual palpation at 11 out of a total of 18 examined tender-point localizations [3]. The exclusion criteria of the GAU-study 2004–2005 were other severe somatic or psychiatric disorders or ongoing exercise therapy supervised by a physical therapist [3].

##### Exclusion criteria

Participants who had deceased, or developed serious physical or psychological disease since they were included in the GAU-study, such as cancer during treatment, stroke with severe physical impairment or schizophrenia.

#### Recruitment

All 166 women received a letter with information about the present study, except four participants who were deceased. The letter was followed up for the 162 participants by telephone for more information about the study and invitation to participate. Those who could not be reached by phone received a second letter with inquiry about new contact information. Eight persons were excluded during the phone call according to exclusion criteria, and 19 declined to participate. Nine could not be reached. A final total of 126 women (76%) participated in the present follow-up.

### Data collection

#### Procedure

The examinations were performed by trained physical therapists who were blinded to previous baseline test results. The examinations took place at rehabilitation centers in the three cities Göteborg, Alingsås and Uddevalla in western Sweden, from April to December 2016. The same measurements that were used at baseline were included in the follow-up examination which lasted about 1.5 h and included a battery of questionnaires, physical examination of tender points [3] and tests of physical function and a standardized interview with questions about duration of pain, employment status, sick leave and disability pension, education, marital status, concomitant disorders and medications.

#### Background data

*Employment* was divided into four categories referring to percentage of full time work, which is defined as 40 h per week.

*Sick leave* and *Disability pension* were categorised as 0, 25, 50, 75% or 100% sick leave/disability pension.

*Marital status* referred to whether the patient lived with another adult or not.

*Education* was divided into three categories referring to years of education since 1st grade: ≤9 years/9–12 years/> 12 years.

*Medications.* Use of analgesics/NSAID and psychotropics (meaning antidepressants and sedatives) was registered as positive when use was regular or as needed.

#### Self-administered questionnaires at baseline and follow-up

##### Pain distribution (0–18)

The localization and distribution of pain were reported in a self-administered pain drawing with 18 predefined body regions [18].

##### The Fibromyalgia Impact Questionnaire (FIQ) (0–100)

The FIQ comprises 10 subscales of disabilities and symptoms, ranging from 0 to 100 [19], and it is validated for a Swedish FM population [20]. A higher total score indicates lower overall health status. The FIQ total score is calculated by the mean of the ten subscales [19]. A total score < 39 is considered to represent mild impairment, ≥39 to > 59 moderate impairment and ≥ 59 severe impairment [21]. The FIQ subscales for Pain intensity *(FIQ pain)* and global fatigue *(FIQ fatigue)* were also applied in the present study. The patients estimated their pain intensity/how tired they had been during the previous week on a VAS, ranging from 0 to 100 mm [19].

##### The Hospital Anxiety and Depression Scale (HADS) (0–21)

The HADS contains 14 statements, rated from 0 to 3. The scores build two subscales for anxiety (HADS-A) and depression (HADS-D), each ranging from 0 to 21, and higher scores indicate a higher degree of distress [22]. .A cut-off score of eight is suggested to indicate possible anxiety or depression [23].

##### Stress and Crisis Inventory (SCI-93) (0–140)

The SCI-93 comprises 35 clinical manifestations of stress. The participants rated to what extent they are interfered by the different symptoms in their daily life on a scale ranging from 0 “not at all” to 4 “very much”. The items include physical and mental sensations. The total score ranges from 0 to 140 and a higher score indicates more stress [24].

##### Short-form 36 (SF-36) (0–100)

The SF-36 is a generic instrument assessing health related quality of life, comprising eight subscales. The subscales build two composite scores, the Physical Component Summary (PCS) and the Mental Component Summary (MCS), ranging from 0 to 100. A higher score indicates better health related quality of life [25].

##### Leisure Time Physical Activity Instrument (LTPAI) (h)

The LTPAI assesses the amount of physical activity during a typical week. The LTPAI total score is the sum of hours for the activities [26].

#### Tests of physical function at baseline

##### Six-minute walk test (6MWT) (m)

The patient was instructed to walk as quickly as she could without running and the baseline values of the distance in metres covered in 6 min was used as a measure of walking capacity in the analyses of predictors of improvement in pain intensity in the present study. The test has shown satisfactory test-retest reliability in a Swedish FM population [27]. The 6MWT was used only in the analyses of predictors of improvement of pain intensity in the present study.

### Statistics

Descriptive statistics are presented as mean, standard deviation (SD) and range (min-max) for continuous variables and as number and percent for categorical variables.

For comparisons over time within the cohort, Wilcoxon’s Signed rank test was used for change over time in continuous variables and Sign test for dichotomous and ordinal categorical variables. Bootstrapped (10000 replicates) 95% confidence intervals were calculated for change over time in continuous variables.

Logistic regression analyses were used to analyze which variables were significant predictors of improvement in pain intensity [28]. Dependent variable was at least 50% improvement in pain intensity as reported on FIQ pain after 10 to 2 years (0 = < 50% improvement or no improvement/1 = ≥50% improvement). Independent variables were baseline values for age, education, BMI, FIQ total score, FIQ pain, HADS-A, HADS-D, SF-36 PCS, SF-36 MCS, LTPAI, 6MWT and group of randomization in previous RCT.

Odds ratios (OR) with 95% confidence intervals and *p*-values are presented.

The independent variables that were associated (*p* < 0.1) with the dependent variable in the univariable logistic regression analyses were included in the multivariable forward stepwise logistic regression analysis. All univariable regression analyses were adjusted for FIQ pain at baseline. Odds ratios (OR) with 95% confidence intervals, p-values and area under the receiving operating curve (AUC) values [29] are presented. AUC-values ranging from 0.7 to 0.8 indicate acceptable goodness of the model, values from 0.8 to 0.9 indicate excellent goodness and over 0.9 indicate outstanding goodness of the model [29]. For comparisons between patients followed-up and lost to follow-up, the Mann Whitney U-test was used for continuous variables, Fisher’s exact test for dichotomous variables and Mantel- Haenszel chi-square test for ordinal categorical variables.

## Results

### Study population

Baseline descriptive data for the 126 patients in the follow-up as well as for the 40 patients who did not participate in the follow-up are presented in Table 1.

**Table 1:** Baseline descriptive data for the 126 patients who participated in the follow-up and the 40 patients who were not followed up, and *p*-values for difference between groups

	Followed up (*n* = 126)	Not followed up (*n* = 40)	*p*-value
	Mean (SD), Min;Max	Mean (SD), Min;Max	
Age, *years*	45.4 (8.6), 22;60	46.3 (9.5), 24;59	0.51
Duration of WP*, years*	10.4 (7.2), 0.3;45	10.6 (7.0), 1.0;30	0.80
Tender points, *n*	13.8 (3.5), 3;18	12.6 (3.4), 6;18	**0.023**
FIQ pain, *(0–100 mm)*	68.3 (18.7), 20;100	71.5 (18.0), 22;100	0.31
Pain distribution, *(0–18)*	13.0 (3.4), 4;18	12.5 (3.4), 5;18	0.38
	n (%)	n (%)	
Education			0.79
≤ 9 years	29 (23%)	9 (23%)	
10–12 years	69 (55%)	20 (50%)	
< 12 years	28 (22%)	10 (25%)	
Work status			0.92
0%	73 (58%)	25 (63%)	
1–49%	14 (11%)	0 (0%)	
50–79%	28 (22%)	9 (23%)	
80–100%	11 (9%)	6 (15%)	
Sick-leave			0.98
0%	66 (52%)	21 (52%)	
25%	5 (4%)	3 (8%)	
50%	17 (13%)	4 (10%)	
75%	2 (2%)	0 (0%)	
100%	36 (29%)	12 (30%)	
Disability pension			0.59
0%	74 (59%)	26 (65%)	
25%	4 (3%)	0 (0%)	
50%	17 (14%)	5 (13%)	
75%	3 (2%)	0 (0%)	
100%	28 (22%)	9 (22%)	
Living with adult	95 (75%)	28 (70%)	0.54
Born outside Sweden	18 (14%)	9 (23%)	0.23
Medications			
Analgetics	87 (69%)	31 (78%)	0.42
Neuroleptica	59 (47%)	15 (38%)	0.36

*WP* Widespread pain, *FIQ* Fibromyalgia Impact Questionnaire Education in Not followed-up: *n* = 39. In bold: *p-*values <0.05

There were no significant differences in baseline values for descriptive data or the primary outcomes pain intensity and distribution of pain, between the participants who participated in the follow-up and the ones who did not, except for the number of tender points (Table 1).

### Change from baseline to follow-up in pain intensity and distribution of pain

There was a significant (*p* < 0.001) improvement in pain intensity (FIQ pain) and distribution of pain at the 10 to 12 year follow-up (Table 2).

**Table 2:** Distributions of pain and other health related variables at baseline, 10 to 12 year follow-up and change from baseline to follow-up. Number and percent of participants that improved are presented for each variable *(n = 121–126)*

	Baseline Mean (SD) Median (Min;Max)	Follow-up Mean (SD) Median (Min;Max)	Change Mean (SD)Median (Min; Max) (95% CI for Mean)^a^	Improved *n* (%)	*p*-value for change
FIQ Pain (0–100) *n = 123*	68.3 (18.7) 70.0 (20; 100)	59.4 (22.0) 63.0 (6; 100)	−9.2 (23.3) −5.0 (−72; 45) (−13.3; −5.0)	75 (61)	**<.0001**
Pain distribution (0–18) *n = 126*	13.2 (3.4) 13.0 (4; 18)	11.1 (4.7) 11.5 (2; 18)	−2.0 (4.2) −2.0 (− 15; 7) (−2.7; − 1.2)	74 (59)	**<.0001**
FIQ total (0–100) *n = 125*	63.5 (16) 64.6 (14; 96)	50.4 (20) 51.6 (10; 96)	− 13.3 (17.9) − 10.0 (− 58; 38) (− 16.4; − 10.2)	93 (75)	**<.0001**
FIQ fatigue (0–100) *n = 124*	78.5 (20.3) 83.0 (15; 100)	71.7 (24.7) 79.0 (0; 100)	−7.0 (21.5) −3.0 (−80; 43) (− 10.8; −3.3)	67 (54)	**0.001**
HADS-D (0–21) *n = 124*	6.8 (3.9) 6.0 (0; 16)	5.8 (4.2) 5.0 (0; 17)	−0.9 (3.9) − 1.0 (− 13; 8) (− 1.6; −0.2)	64 (52)	**0.020**
HADS-A (0–21) *n = 124*	8.2 (5.2) 7.0 (0; 20)	7.7 (4.7) 7.0 (0; 18)	−0.5 (4.4) − 1.0 (−12; 10) (−1.3; 0.3)	65 (52)	0.32
SF36 PCS (0–100) *n = 121*	29.7 (8.2) 29.3 (11; 50)	33.9 (10.2) 33.4 (10; 58)	4.0 (10.2) 4.0 (−15; 34) (2.2; 5.8)	76 (63)	**< 0.001**
SF36 MCS (0–100) *n = 123*	39.5 (13.4) 41.2 (11; 67)	39.6 (14.7) 43.0 (0; 68)	0.2 (14.5) 1.4 (−67; 30) (− 2.4; 2.7)	81 (66)	0.42
SCI-93 (0–140) *n = 122*	76.3 (24.0) 76.3 (26; 132)	69.4 (26.2) 66.0 (6; 128)	−6.5 (20.8) −3.5 (−82; 33) (−10.3; −2.8)	68 (56)	**0.005**
LTPAI (h) *n = 122*	5.0 (3.9) 4.0 (0; 23)	5.5 (5.4) 4.0 (0; 34)	0.5 (5.6) 0.0 (−11; 27) (−0.5; 1.5)	51 (42)	0.67

^a^Bootstrapped (10,000 replicates) 95% CI for Mean. *FIQ* Fibromyalgia Impact Questionnaire, *HADS-D/HADS-A* Hospital Anxiety and Depression Scale – Depression/Anxiety, *SF-36* Short-form 36, *PCS* Physical Component Summary, *MCS* Mental Component Summary, *SCI-93* Stress and Crisis Inventory, *LTPAI* Leisure Time Physical Activity Instrument In bold: *p*-values <0.05.

In pain intensity (FIQ Pain), 11 (9%) of the women showed an individual moderate improvement of 30 to 50% and 20 women (16%) showed a substantial improvement of *≥*50% at the follow-up as compared to baseline. Nine (7%) had a moderate worsening of pain intensity of 30 to 50% and 7 (6%) had a substantial worsening of *≥*50%. The remaining 76 women (60%) remained either unchanged, < 30% improvement or < 30% worsened. Missing data was found for three women (2%) in FIQ pain, why the change in pain intensity was calculated for 123 of the 126 women.

### Change from baseline to follow-up in other health related aspects

The 126 women improved on group-level over 10 to 12 years in overall health as assessed with the FIQ total score (Table 2). The distribution of proportions in categories of impairment [21] at baseline and the 10 to 12 year follow-up is presented in Table 3.

**Table 3:** Level of impairment based on FIQ total score for the 126^a^ women participating in the 10 to 12 year follow-up

	Baseline *n* (%)	Follow-up *n* (%)
Mild impairment (< 39 p)	11 (9)	43 (34)
Moderate impairment (≥39 to < 59 p)	35 (28)	40 (32
Severe impairment (≥59 p)	79 (63)	42 (33)

^a^1 missing value was found both at baseline and at follow-up for FIQ total score

The 126 women improved significantly in Global fatigue (FIQ fatigue) (*p* = 0.001), depression (HADS-D) (*p* = 0.021), the physical component of health related quality of life (SF-36-PCS) (*p* < 0.001) and symptoms of stress (SCI-93) (*p* = 0.005) at the 10 to 12 year follow-up as compared to baseline (Table 2). There were no significant change in anxiety (HADS-A), the mental component of health related quality of life (SF-36 MCS) or the level of physical activity (LTPAI) at the 10 to 12 year follow-up (Table 2).

### Predictors of ≥50% improvement in pain intensity

Significant univariable predictors of at least 50% improvement in FIQ pain were baseline values of FIQ total, HADS depression, SCI-93 and SF-36 MCS (Table 4). These four variables together with FIQ pain baseline were included in the multivariable forward stepwise logistic regression analysis. The result showed that SCI-93 (OR 0.69, 95% CI: 0.53–0.89, *p* = 0.004) and FIQ pain (OR 1.49, 95% CI: 1.08–2.07, *p* = 0.016) contributed to the strongest model (AUC: 0.730) for predictors of a reduction in pain intensity with at least 50%. Lower symptoms of stress and higher pain intensity at baseline predicted higher probability of reporting 50% less pain intensity after 10 to 12 years as compared to baseline.

**Table 4:** Univariable logistic regression analyses of predictors of a reduction in FIQ pain *≥*50% after 12 years. Odds ratios with 95% confidence intervals and p-values, adjusted for FIQ pain at baseline *(n = 123*^a^*)*

*Baseline values*	*Dependent variable ≥* 50% reduction in FIQ pain	
	OR (95% CI)	*p*-value
Age, *per 10 years*	1.00 (0.95–1.06)	0.85
Education, *1–4*	0.97 (0.47–2.03)	0.95
Body Mass Index	0.95 (0.86–1.05)	0.32
FIQ total*, per ten units*	0.66 (0.45–0.96)	**0.031**
FIQ pain*, per ten mm*	1.19 (0.91–1.57)	0.21
HADS anxiety, *0–14*	0.93 (0.84–1.03)	0.14
HADS depression*, 0–14*	0.85 (0.74–0.99)	**0.030**
SF-36 PCS, *per ten units*	0.70 (0.35–1.41)	0.31
SF-36 MCS, *per ten units*	1.87 (1.18–2.97)	**0.008**
SCI-93, *per ten units*	0.79 (0.64–0.98)	**0.032**
LTPAI, *hours*	0.97 (0.85–1.11)	0.67
6 min walk test, *meter*	1.00 (0.99–1-01)	0.97
Randomization in previous RCT	1.50 (0.57–3.99)	0.42

*p*-values less than 0.05 in bold

*FIQ* Fibromyalgia Impact Questionnaire, *HADS* Hospital Anxiety and Depression Scale, *SF-36* Short-form 36, *PCS* Physical Component Summary, *MCS* Mental Component Summary, *SCI-93* Stress and Crisis Inventory, *LTPAI* Leisure Time Physical Activity Instrument

^a^Data for 123 women were included in the analyses of improvement in pain intensity (FIQ pain), due to missing values. For HADS anxiety, HADS depression, SF-36 PCS, SF-36 MCS and SCI-93 the number of cases included in analyses were 120–122

## Discussion

The present study assessed the course of symptoms over 10 to 12 years in a cohort of women with FM and CWP as well as predictors of substantial improvement in pain intensity.

The women were found to improve over time in pain intensity. The mean improvement for pain intensity for the study population was 9 mm on a 100 mm VAS which correspond to a 13% change from baseline on group-level. This result is in line with a previous follow-up study in which an improvement of 11 mm was found over 3 years in patients with FM [8]. On the contrary the present results are contradictory to the findings of an 11 year follow-up in FM by Wolfe et al. which did not show a clinically relevant improvement in pain, fatigue or overall health status [10]. The divergent results could be due to different study settings; primary care in the present study versus secondary or tertiary care in the Wolfe study.

An individual change in pain intensity of 30 to 50% from baseline has been suggested to reflect a moderate clinically important improvement for patients with chronic pain [14, 15], which in the present study was found for 9% of the women. A substantial improvement in pain intensity is considered to be reflected by an individual change of at least 50% [14, 15] which was achieved by 16% of the women in the present study. These results are in line with a previous 5 year follow-up study in FM, which found a moderate improvement in pain intensity for 10% and a substantial improvement for 12% of the population over time [15]. However, another follow-up study found that 36% of patients with FM reported at least 30% improvement in pain intensity after 3 years [8]. The suggested cut-offs for level of improvement in pain intensity were based on clinical trials [14]. In the present study the limits were used as a guidance of how to interpret the clinical relevance of the change in pain intensity in a cohort over time, why the conclusion should be interpreted with caution.

The distribution of pain was found to be improved over 10 to 12 years in the present study population with a mean reduction of two pain sites. This improvement in pain spread corresponds to previous findings in general populations which have shown that some persons can recover from CWP over time [8, 9].

Lower levels of stress symptoms at baseline assessed with the SCI-93 and higher pain intensity predicted higher chance of substantial improvement in pain intensity after 10 to 12 years in the present study. The SCI-93 reflects the severity of clinical manifestations of stress related to the autonomous nervous system. Physiological stress mechanisms have also been suggested to play a role in the maintenance of widespread pain in FM [30, 31], which supports the findings in the present study. A high degree of clinical stress symptoms in women with FM and CWP might be related to the amount of neurobiological aberrations which have been observed in patients with chronic pain in the hypothalamic-pituitary-adrenal (HPA) axis and the noradrenaline-sympathetic system, components of the human stress response [30, 31]. Exposure to stress over a long period of time may trigger dysfunctional biological and psychological stress mechanisms which could have a negative impact on the patient’s health and possibilities to recovery [32]. The results of the present study underlines the importance of development of treatments and strategies in health care to reduce stress in women with FM and CWP.

In the univariable analyses, pain intensity at baseline was not significantly associated with ≥50% improvement in pain intensity after 10 to 12 years. However, in the multivariable analysis, lower stress levels and higher pain intensity at baseline were found to predict substantial improvement in pain intensity. The fact that patients with higher levels of pain are more likely to improve in pain over time could partly be due to regression to the mean. The findings in the present study enhances the assumption that stress level is an important aspect to take into consideration in women with FM and CWP, especially for patients with more severe pain. The AUC-value for the model was 0.730, indicating acceptable goodness of the model, and that other factors not found in the present study may play a role in substantial improvement in pain intensity over time.

Besides clinical symptoms of stress, the univariable models in the present study showed that mental aspects of health such as symptoms of depression and the SF-36 MCS may play an important role in predicting chances of substantial pain reduction after 10 to 12 years. Physical aspects of health such as SF-36 PCS, level of physical activity and walking capacity at baseline were not found to be associated with substantial improvement in pain intensity after 10 to 12 years. However, although physical function did not predict improvement in pain over time in the present study, physical exercise has been found to contribute to improvements in all core symptoms of FM and CWP in intervention studies [33–36]. It is thus crucial to promote physical activity and physical function in treatments for patients with FM and CWP in order to improve general health and reduce the risk for concomitant disorders related to a sedentary life-style.

For FIQ total score the mean improvement from baseline to follow-up corresponds to a change of 20% which is higher than the minimal clinically important change for the FIQ total score in women with FM which has been suggested to be around 14% [21] . Although this interpretation is based on intervention studies and a shorter time period [21] it could be used as a guidance for interpretation of the clinical relevance of change in overall health in the present study.

The number of patients with severe impairment [21] has halved in the present study at the 10 to 12 year follow-up. Still, over 30% of the women experienced severe impairment after 10 to 12 years which underlines the importance of finding adequate methods to support these patients over time.

Small but statistically significant improvements over time was also found in the present study for other important aspects of health such as fatigue, depression, symptoms of stress and physical component of health related quality of life. These findings are in line with the results of a previous five-year follow-up reporting small improvements in fatigue, depression and overall health [12]. However, another follow-up study showed no improvement in fatigue and depression in women with FM over time [11]. Discrepancies in results between the studies might be due to the use of different questionnaires or differences in population characteristics.

Although the patients in the present study improved over time in many aspects of health, their mean levels of pain, fatigue and health related quality of life still reflect severe impairment. Symptoms of anxiety and mental aspects of health related quality of life did not change significantly over time.

The total amount of time spent on physical activity in leisure time did not change significantly over 10 to 12 years in the present study, the mean value being around 5 h/week at both examinations, including activities on low and moderate intensity. This result reflects the difficulties of being physically active for patients with chronic pain.

The follow-up rate in the present study was high, 76%, and the patients who were followed up did not differ significantly in baseline values from the ones who did not participate in the follow-up, except for a slightly higher number of tender points.

### Limitations

The present follow-up study aimed to assess the course of symptoms over time in women with FM or CWP. The population participated 10 to 12 years earlier in an RCT comprising an intervention of pool exercise and patient education [17]. Therefore, the cohort might consist of participants with a positive attitude towards physical exercise, which has to be taken into consideration when interpreting the generalizability of the results. The impact of the exercise program is considered to be negligible after 10 to 12 years and previous randomization were not found to be associated with improvement in pain intensity in the univariable analyses of predictors. The participants were recruited from primary health care centers in which many patients with chronic pain are offered participation in patient education, why the population in the present study is considered to reflect average patients with FM and CWP found in Swedish primary health care.

Symptoms in FM and CWP fluctuate from day to day and another limitation of the present study is that the 10 to 12 year follow-up was performed at only one occasion. A part of the small but significant improvement found in pain intensity and other symptoms could possibly be due to a natural regression to the mean over time. Also, other variables than baseline symptoms and characteristics might have influenced the course of symptoms over time, such as concomitant disorders, different treatments or major life changes in work and social relations. No information of pharmacological or non-pharmacological treatments or other life changes during the study period were gathered since it was not likely that the participants could recall accurate information after 10 to 12 years.

## Conclusions

A majority of women with FM and CWP appear to have a positive course of pain and other symptoms on group-level over 10 to 12 years, which should be communicated to these patients in health care. In the present study, the women were found to improve in pain intensity with 13% over 10 to 12 years. A substantial improvement in pain intensity was achieved by 16% of the women. Also the pain spread, overall health, fatigue, symptoms of depression, stress and physical component of health related quality of life were found to improve over time, however the mean improvements were small.

Lower levels of clinical stress symptoms and higher pain intensity predicted higher chances of substantial improvement in pain intensity 10 to 12 years later. Therefore, stress is recommended to be assessed and taken in to account in the planning of treatments of patients with FM and CWP.

## Data Availability

The datasets used and/or analyses during the current study are available from the corresponding author on reasonable request.

## Abbreviations

6MWT: 6 min walk test
AUC: Area under the receiving operating curve
BMI: Body Mass Index
CWP: Chronic Widespread Pain
FIQ: Fibromyalgia Impact Questionnaire
FM: Fibromyalgia
HADS: Hospital Anxiety And depression Scale
SCI-93: Stress and Crisis Inventory
LTPAI: Leisure Time Physical Activity Instrument
MCS: Mental Component Summary
OR: Odds Ratio
PCS: Physical Component Summary
RCT: Randomised Controlled Trial
SF-36: Short-Form 36
VAS: Visual Analogue Scale
